# Associations Between Walking in the Third Trimester of Pregnancy and Maternal Mental Health During the COVID-19 Pandemic

**DOI:** 10.3390/ijerph22101538

**Published:** 2025-10-08

**Authors:** Angélique Brun, Stephanie-May Ruchat, Sophie Chaput-Langlois, Linda Booij, Raphaëlle Giac, Katherine Séguin, Andréanne Bernier, Anne-Sophie Morisset, Isabelle Boucoiran, Cathy Vaillancourt, Sarah Lippé, Catherine M. Herba

**Affiliations:** 1 Department of Psychology, Université du Québec à Montréal (UQAM), Montreal, QC H2X 3P2, Canada; brun.angelique@courrier.uqam.ca (A.B.); chaput-langlois.sophie@courrier.uqam.ca (S.C.-L.); giac.raphaelle@courrier.uqam.ca (R.G.); seguin.katherine.2@courrier.uqam.ca (K.S.); 2 CHU Sainte-Justine Azrieli Research Center, Montreal, QC H3T 1C5, Canada; linda.booij@mcgill.ca (L.B.); isabelle.boucoiran@umontreal.ca (I.B.); sarah.lippe@umontreal.ca (S.L.); 3 Department of Physical Activity Sciences, Trois-Rivières, Université du Québec à Trois-Rivières (UQTR), Trois-Rivieres, QC G9A 5H7, Canada; stephanie-may.ruchat@uqtr.ca; 4 Department of Psychiatry, Université McGill, Montreal, QC H3A 1G1, Canada; 5 Eating Disorders Continuum & Research Centre, Douglas Mental Health University Institute, Montreal, QC H4H 1R3, Canada; 6 Département des Sciences de la Santé, Université du Québec en Abitibi-Témiscamingue, Rouyn-Noranda, QC J9X 5E4, Canada; andreanne.bernier@uqat.ca; 7 École de Nutrition, Université Laval, Québec, QC G1V 0A6, Canada; anne-sophie.morisset@fsaa.ulaval.ca; 8 Axe Endocrinologie et Néphrologie, Centre de Recherche du CHU de Québec, Université Laval, Québec, QC G1E 6W2, Canada; 9 Centre Nutrition, Santé, et Société (NUTRISS), Institut sur la Nutrition et les Aliments Fonctionnels (INAF), Université Laval, Québec, QC G1V 0A6, Canada; 10 Department of Psychology, Université de Montréal, Montreal, QC H2V 2S9, Canada; 11 INRS-Centre Armand-Frappier Santé Biotechnologie, Laval, QC H7V 1B7, Canada; cathy.vaillancourt@inrs.ca; 12 Centre de Recherche du CIUSSS-du-Nord-de-l’Île-de-Montréal, Montreal, QC H4J 1C5, Canada

**Keywords:** COVID-19, pregnancy, prenatal mental health, physical activity, walking, third trimester

## Abstract

Prenatal physical activity (PA) has well-established benefits for maternal mental health. However, PA levels are generally low among pregnant individuals and were even lower during the COVID-19 pandemic. Since walking is the most popular form of prenatal PA, we aimed to examine associations between walking in the third trimester of pregnancy and mental health symptoms of depression, anxiety, pregnancy-related anxiety and perceived stress during the pandemic. Relevant pandemic-related factors (e.g., COVID-19 waves, population density) associated with walking were also studied. Pregnant individuals were recruited across Quebec (Canada) between October 2020 and September 2022, as part of the Resilience and Perinatal Stress during the Pandemic (RESPPA) study. Analyses were conducted on data collected via online questionnaires during the third trimester (*n* = 1086). Results revealed that higher levels of walking were significantly associated with lower symptoms of generalized anxiety (β = −0.06, *p* = 0.035), and perceived stress (β = −0.07, *p* = 0.007). Living in a more densely populated area, living with fewer children at home and having a university degree were associated with higher levels of walking. Those who completed their questionnaire in the second pandemic wave also reported higher levels of walking. Our results highlight the potential of walking in the third trimester to support maternal mental health.

## 1. Introduction

Pregnancy represents a vulnerable period for mental health difficulties due to the many social, hormonal and psychosocial changes entailed [1,2,3,4]. This vulnerability was heightened during the COVID-19 pandemic, since symptoms of psychological distress were found to be elevated for pregnant individuals [5,6]. Indeed, three large Canadian prospective studies indicated higher symptoms of depression and anxiety among Canadian pregnant individuals following the onset of the pandemic compared to the pre-pandemic period [7,8,9]. During the pandemic, prevalence rates of clinically elevated symptoms of prenatal depression and anxiety were reported to be 25.6% and 30.5%, respectively [6]. Most studies conducted in the context of the pandemic have focused on symptoms of prenatal depression and/or anxiety [5]. However, it is important to also consider other aspects of maternal psychological distress, such as perceived stress and pregnancy-related anxiety. Indeed, stress during pregnancy reduces the functioning of the maternal immune system, rendering pregnant individuals more vulnerable to infections, diseases and complications [10]. In addition, some studies consider pregnancy-related anxiety to be a better predictor of child development than generalized anxiety [11,12]. Further, the various manifestations of prenatal maternal psychological distress have been associated with poorer postnatal mental health [2] as well as poorer child development (e.g., negative affectivity, difficult temperament) [13]. Prenatal maternal mental health is therefore a public health priority [14,15]. It is essential to gain a better understanding of factors that can be leveraged to support and improve maternal mental health during pregnancy, particularly in the context of events that can accentuate psychological distress, such as a pandemic.

Several studies addressing prenatal mental health highlighted prenatal physical activity (PA) as a protective factor [16,17]. Meta-analyses have shown that regular practice of prenatal PA reduces the odds of developing prenatal depression by 67% and the severity of prenatal depressive symptoms (Standardized Mean Difference (SMD) = −0.38, small effect size) [17]. Interestingly, during the COVID-19 pandemic, pregnant individuals who met the recommendations for prenatal PA (i.e., 150 min/week of moderate-to-vigorous PA) had significantly lower levels of anxiety and depression than those who did not [18]. Despite the mental health benefits, as well as other health benefits of prenatal PA [19], it is well established that pregnant individuals are not active enough [20,21] and that PA levels decrease throughout pregnancy, with the most pronounced decline occurring during the third trimester [20,22,23].

Walking is the most popular form of prenatal PA and can be recommended throughout all stages of pregnancy since it is accessible, affordable, generally safe and easy to integrate into daily life [24]. Research has focused on its specific influence on prenatal physical health and has shown that walking provides several maternal health benefits, such as a decreased risks of preeclampsia, excessive weight gain, and depression. However, only a few studies have examined associations between walking and specific indices of maternal mental health [24,25,26]. Yet, these studies on walking have been limited since they had small sample sizes, tended to focus on depression without considering other mental health symptoms such as pregnancy-specific anxiety or perceived stress, and were not conducted in the context of COVID-19. Because it is safe, inexpensive, and widely accessible, walking represents a promising public health strategy, as it could be implemented at the population level to support both maternal physical and mental health. Understanding its associations with various aspects of maternal mental health is therefore particularly important, especially during periods of heightened vulnerability.

Several factors, such as environmental (e.g., weather, season, access to sport facilities, cycling paths and parks) and sociodemographic (e.g., age, number of children, socioeconomic status) factors, are known to influence prenatal PA practice, including walking [23,27,28]. However, much less is known about whether such environmental factors were also associated with walking during pregnancy in the context of the COVID-19 pandemic. Furthermore, certain factors related to the pandemic might be associated with walking, such as the pandemic wave and associated restrictions or the population density of the individual’s neighbourhood. It is plausible that pregnant individuals would walk less if they live in larger, more densely populated urban centers, with reduced access to green spaces and greater risk of acquiring COVID-19 infection [29]. On the other hand, those living in more rural, or less densely populated areas, might find it harder to walk due to larger distances between locations or less safe walking conditions (e.g., lack of sidewalks) [30,31]. However, to the best of our knowledge, previous work has not examined the contributions of such relevant factors that could be associated with walking in late pregnancy, such as (1) the wave of the COVID-19 pandemic; (2) population density; and (3) COVID-19-related concerns (e.g., worries about catching COVID-19 or risk of infection for the unborn baby).

The present study, conducted in the context of the COVID-19 pandemic, addressed these gaps through the following aims to examine (1) the associations between walking in the third trimester of pregnancy and complementary yet different indices of maternal mental health (i.e., symptoms of depression, generalized anxiety, perceived stress and pregnancy-related anxiety); and (2) factors associated with reported walking levels in the third trimester of pregnancy.

## 2. Materials and Methods

### 2.1. Participants and Procedure

We addressed our research questions within the longitudinal study *Resilience and perinatal stress during the pandemic* (RESPPA). Between October 2020 and September 2022 (i.e., spanning the second to the seventh wave of the COVID-19 pandemic [32]), pregnant individuals or mothers who had given birth in the previous three months were recruited across different regions of the Canadian province of Quebec. We did not collect any data during the first wave of COVID-19. To facilitate recruitment, either clinical staff from partner hospital sites mentioned the study to pregnant individuals or participants were recruited through advertisements on social media platforms (e.g., Facebook, Instagram). Inclusion criteria included residing in the province of Quebec (Canada), being over 18 years of age, ability to speak and write in French and/or English and having a viable pregnancy. Participants were invited to complete a 60-min self-report questionnaire online via *LimeSurvey*, available in French or English, depending on the participant’s preference. Data were collected at multiple time points throughout the perinatal period. However, the current substudy has a cross-sectional design, and analyses focused exclusively on individuals who answered both questions on walking during the third trimester of pregnancy, resulting in a sample of 1086 pregnant individuals. Participation was voluntary and informed consent was obtained prior to questionnaire completion. This multicenter project was approved by the ethics committee of the central site (CHU Sainte-Justine), the partner sites, and at the Université du Québec à Montréal.

### 2.2. Measures

#### 2.2.1. Walking Levels

Walking was assessed using two questions from the short version of the Pregnancy Physical Activity Questionnaire (PPAQ) [33]. The PPAQ is a widely used, reliable and validated instrument [34] available in both English [35] and French [36]. For the purposes of this study, we focused on the two questions pertaining to walking that were included in the version administered: the duration and intensity devoted to slow walking and fast uphill walking over the last 3 months. Individuals had to select one of the six options for walking duration, ranging from “None” to “3 or more hours/week”. The duration of each activity (i.e., slow walking and fast uphill walking) was then multiplied by its intensity, estimated in metabolic equivalents (METs) as reported in the Adult PA Compendium [37]. As such, an intensity of 3.2 METs were assigned to slow walking and 6.5 METs to fast uphill walking. Energy expenditure for each walking type was calculated using the following formula: Duration * Intensity (either 3.2 or 6.5). The resulting values for both walking items were then summed to produce a total score of walking (METs min/week).

#### 2.2.2. Prenatal Maternal Mental Health Symptoms

Depressive symptoms were assessed using the Edinburgh Postnatal Depression Scale (EPDS) [38], which consists of 10 items rated on a 4-point Likert scale. Total scores range from 0 to 30, with a cutoff score of 13 and above commonly used to indicate clinically relevant levels of depressive symptom [39]. Generalized anxiety symptoms were assessed using the Generalized Anxiety Disorder-7 (GAD-7) [40]. Items are rated on a 4-point Likert scale, with responses ranging from “Not at all” (coded as 0) to “Nearly every day” (coded as 3), with total scores varying from 0 to 21. Anxiety severity on the GAD-7 can be categorized as mild, moderate, or severe, corresponding to scores of 5, 10, and 15, respectively [40]. Pregnancy-related anxiety was evaluated using the short version of the Pregnancy-Related Anxiety Questionnaire (PRAQ-R2), which includes 10 items [11]. These items assess concerns about childbirth, the health of the unborn baby or changes in physical appearance, using a 5-point Likert scale ranging from “Absolutely not relevant” (coded as 1) to “Very relevant” (coded as 5). Total scores range from 10 to 50, with higher scores indicating more severe symptoms. A threshold of 34 or above has been used in previous research to indicate clinically significant levels of pregnancy-specific anxiety [41]. Finally, to measure perceived stress, the Perceived Stress Scale (PSS) [42] was administered. It comprises 10 items assessing the extent to which life events are perceived as threatening, unpredictable or uncontrollable. Responses are scored on a 5-point Likert scale, ranging from “Never” (coded as 0) to “Very often” (coded as 4), with higher scores indicating greater levels of perceived stress. Perceived Stress scores can be interpreted as follows: 0–13 indicating low stress, 14–26 indicating moderate stress, and 27–40 indicating high stress [43,44].

A section of the RESPPA questionnaires was designed to measure concerns about COVID-19. Co-authors Herba and Booij developed a short questionnaire to obtain information on the impact of COVID-19 on mothers’ well-being. For this investigation, six items pertaining to pandemic-related health concerns were evaluated, with questions such as: “In the context of the COVID-19 pandemic, over the past two weeks, how worried were you about becoming infected with COVID-19?” Responses were rated on a 7-point Likert scale, ranging from (1 = “Not at all” to 7 = “Very much”). Higher scores reflected higher levels of concerns.

The internal consistency for all measures was good to excellent, with the following Cronbach’s alphas: EPDS: α = 0.87; GAD-7: α = 0.90; PRAQ-R2: α = 0.84; PSS: α = 0.89, and concerns about COVID-19: α = 0.86. For each measure, items were summed to produce a total score reflecting symptoms of depression, generalized anxiety, perceived stress, pregnancy-related anxiety, and concerns about COVID-19.

#### 2.2.3. Covariates

A number of potential covariates were considered, as they could be linked to the central variables of interest. The online questionnaire included questions about sociodemographic indices (e.g., family income, ethnicity, education, number of children at home, approximate location of residence, age of the mother in the third trimester, first pregnancy or not). Additional questions on the mother’s perception of partner support and the presence or absence of pregnancy-related complications were also included. For partner support, mothers responded to four questions such as: “To what extent do you feel supported by your current spouse (partner) in the household chores?”, with an 11-point Likert scale (0 = “Not at all” to 10 = “Totally”) [45]. Higher scores indicated higher levels of perceived partner support. Examples of pregnancy-related complications included preeclampsia, gestational diabetes mellitus, placenta previa and pregnancy-induced hypertension. This question was transformed into a dichotomous variable (i.e., the absence or presence of at least one complication).

The date of questionnaire completion was used to infer the season of completion (e.g., winter, summer, etc.), as well as the pandemic wave at the time of completion. The COVID-19 pandemic wave was based on the timeline made available by the Quebec National Institute of Public Health [32]. A continuous variable capturing the population density of each Forward Sortation Area (FSA)—a geographical unit represented by the first three characters of the postal code in Canada—was used. The variable was calculated by Statistics Canada with data from the 2021 Census and derived from the 2021 Census Boundary File [46], a publicly available data file under an Open Government License. Density represents the number of people living in a given FSA divided by its surface area (in km^2^). The Province of Quebec is divided into 415 FSA and the RESPPA participants lived in 382 of them (92%).

Dichotomous variables were coded as follows: first pregnancy (no = 1, yes = 2); pregnancy complications (no = 0, yes = 1). Number of children at home, partner support, maternal age in the third trimester, and population density were treated as continuous variables. Dummy variables were used to include categorical variables in the analyses (e.g., COVID-19 waves, education level, and season).

### 2.3. Statistical Analysis

Descriptive statistics and preliminary correlation analyses were conducted in IBM SPSS Statistics version 29 software [47]. Main analyses were performed using Mplus 8.11 software [48]. A path analysis was conducted to examine associations between walking in the third trimester and each of the four indices of prenatal mental health (Aim 1). To address the second objective, examining factors associated with walking during the third trimester, a multiple linear regression was performed (Aim 2). Non-normality was adjusted using a robust maximum likelihood estimator (MLR). Among the 1086 participants, some data were missing for mental health variables (from 0.01 to 1.10%) and for covariates (from 0 to 5.2%) due to occasional item non-response. To account for missing data, multiple imputations within the Mplus software were conducted, using 50 imputed datasets and 1000 iterations.

A path analysis was performed examining associations between reported walking levels (independent variable) and each of the four prenatal mental health indicators (dependent variables). This model allowed us to examine associations with the four correlated indices of maternal mental health while reducing the risk of inflated Type 1 error due to multiple testing. An initial unadjusted model was tested to examine the unique contribution of walking. Then, a second model was tested, adjusted for the following covariates: (1) season at the time of questionnaire completion, (2) COVID-19 wave at the time of questionnaire completion, (3) pregnancy complications, (4) first pregnancy or not, (5) education level, (6) perceived partner support, (7) age of the mother in the third trimester of pregnancy and (8) population density. Covariates were selected based on prior literature and/or significant associations—identified through preliminary correlation analyses—with maternal mental health indices. Variables such as perceived partner support and education level were also found to be associated with walking levels and were considered as potential confounding variables in the model. The variables ‘number of children’ and ‘first pregnancy’ were highly correlated (*r* = 0.70). Therefore, only the ‘first pregnancy’ variable was included with the other covariates, as it was the most strongly correlated with the four indices of prenatal maternal mental health. Dummy variables were used to include seasons (winter as the reference group), COVID-19 waves (waves 6–7 combined as the reference group, since they were the least restrictive in terms of government measures [32]), and education level (high school diploma or less as the reference group).

For the multiple linear regression analysis, walking levels were used as the outcome variable. Based on the previous literature cited, the following relevant factors were included in the model as independent variables: COVID-19 waves and concerns, education level, age of the mother, population density, number of children at home, perceived partner support, and pregnancy complications. To avoid multicollinearity, the variable ‘number of children at home’ was prioritized over ‘first pregnancy’ in this second analysis, as it showed a stronger association with walking levels. Education level and COVID-19 waves were included in the regression using dummy variables, as in the path analysis; however, wave 2 was used as the reference group for COVID-19 waves. Preliminary analysis using an analysis of variance showed significantly higher levels of walking during the second wave of COVID-19, which is why it was chosen as the reference group. Preliminary analyses using an analysis of variance also found no significant links between the season and walking in the third trimester, which is why season was not included in the model.

For the path analysis and the multiple linear regression, standardized estimates (β) were reported. Cohen’s (1988) benchmarks [49] were used to interpret effect sizes: 0.10 = small, 0.30 = medium, 0.50 = large. Two-tailed *p*-values < 0.05 were considered statistically significant.

## 3. Results

### 3.1. Descriptive Statistics

Most participants (>55%) in the sample were between 28 and 35 years of age (*Mage =* 31.32 years, *SD =* 4.19), reported high levels of education (i.e., university diploma), and elevated annual family income (i.e., >$100,000 CAD). Nearly all participants (>95%) identified as White and lived with a partner. More than half (>54%) were experiencing their first pregnancy. Most participants (>65%) completed their questionnaire after the fifth wave of the COVID-19 pandemic (i.e., between 5 December 2021–12 March 2022). For more details, see Table 1.

The average score for slow walking was slightly higher than the average score for fast uphill walking. The average scores expressed in METs-min/week indicate that the pregnant individuals in our sample reported on average between 1 and 2 h of slow walking, and between 30 min and 1 h of fast uphill walking, on a weekly basis. Based on the cutoffs presented earlier, 18.1% (EPDS scores ≥ 13) and 11.8% (PRAQ-R2 scores ≥ 34) of pregnant individuals reported clinically significant symptoms of depression and pregnancy-related anxiety, respectively. Additionally, 14.2% (GAD-7 scores ≥ 10) reported moderate to severe generalized anxiety symptoms, and 27.9% (PSS scores ≥ 20) reported moderate to high levels of perceived stress. For more details on the respective distributions (e.g., mean, standard deviation) of the dependent variables, see Table 2.

### 3.2. Associations Between Walking Levels and Maternal Mental Health Symptoms

**Figure 1:**
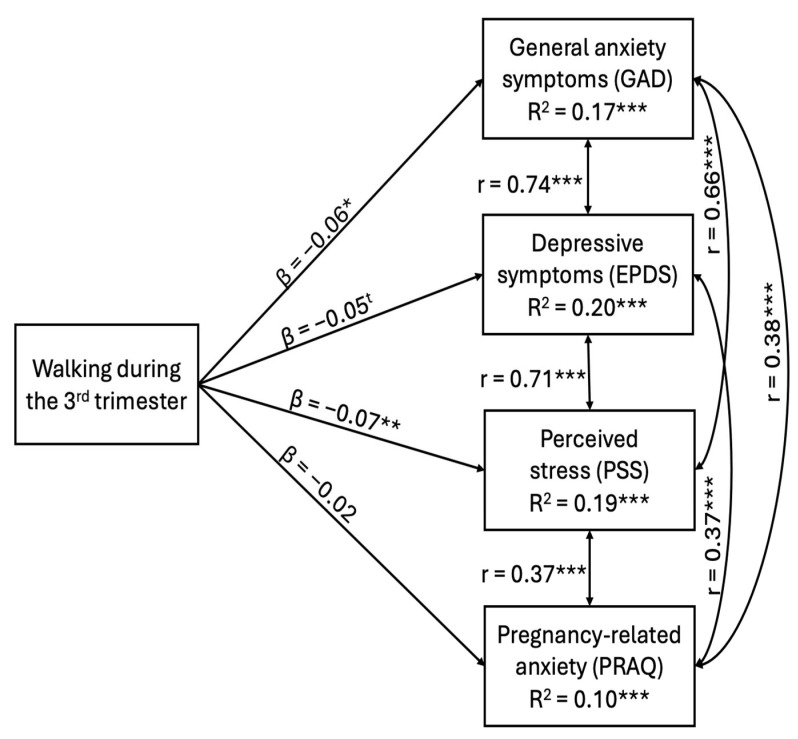
Main findings from the path analysis: Associations between walking levels and prenatal mental health indicators (adjusted Model). Note. * *p* < 0.05, ** *p* < 0.01, *** *p* < 0.001. Two-tailed *p*-values < 0.05 were considered statistically significant. The symbol t indicates a statistical trend, corresponding to a *p*-value between 0.05 and 0.10.

The path analysis revealed that a higher level of walking in the third trimester was significantly associated with lower symptoms of generalized anxiety (β = −0.11, *p* < 0.001), depression (β = −0.11, *p* < 0.001), and perceived stress (β = −0.13, *p* < 0.001), as indicated by Model 1 (unadjusted). When covariates were added (Model 2), a higher level of walking in the third trimester remained significantly associated with lower symptoms of generalized anxiety (β = −0.06, *p* = 0.035) and perceived stress (β = −0.07, *p* = 0.007). Standardized β-coefficients indicated small effect sizes [49]. The negative association between walking and maternal depression symptoms became a non-significant trend in Model 2 (β = −0.05, *p* = 0.098). There was no significant association between walking and pregnancy-related anxiety symptoms in the third trimester in the unadjusted (Model 1) (β = −0.00, *p* = 0.917) or the adjusted (Model 2) (β = −0.02, *p* = 0.508) models. Figure 1 illustrates the main findings regarding the associations between walking levels and the four prenatal mental health indicators, after adjusting for covariates.

Among the covariates considered, perceived partner support showed the highest standardized β-coefficients for all prenatal mental health indices. Thus, greater perceived partner support was associated with lower symptoms of generalized anxiety (β = −0.27, *p* < 0.001), depression (β = −0.34, *p* < 0.001), perceived stress (β = −0.33, *p* < 0.001), and pregnancy-related anxiety (β = −0.18, *p* < 0.001). As reported by the R2, Model 2 explained 17% of the variance in generalized anxiety symptoms, 20% of the variance in depressive symptoms, 19% of the variance in perceived stress symptoms and 10% of the variance in pregnancy-related anxiety symptoms. For more details, see Table 3.

Based on these results, we aimed to examine whether fast uphill walking was driving the observed associations, rather than walking in general. We therefore conducted an additional path analysis. The results are presented in the Supplementary Materials section. We used the same analytical strategy as previously, but rather than using the total walking score, we included two predictors—the slow-walking variable score (in METs-H/week) and the fast-walking variable score (in METs-H/week)—in the same models (Model 1 and Model 2). The four mental health indices remained the dependent variables. The results of this path analysis indicated that higher levels of slow walking were associated with lower levels of generalized anxiety (β = −0.11, *p* < 0.001), depression (β = −0.11, *p* < 0.001), perceived stress (β = −0.10, *p* = 0.002), and pregnancy-related anxiety (β = −0.09, *p* = 0.006) (Model 1). In the same model, higher levels of fast uphill walking were associated with higher levels of pregnancy-related anxiety (β = 0.07, *p* = 0.021), but no associations were observed between fast uphill walking levels and the other three mental health indices. After inclusion of control variables in Model 2, higher levels of slow walking remained significantly associated with lower symptoms of generalized anxiety, depression, perceived stress, and pregnancy-related anxiety, with small effect sizes. No associations were observed between fast walking levels and any of the four mental health indices in Model 2 (see Table S1, Supplementary Materials section).

### 3.3. Factors Associated with Walking Levels

The COVID-19 pandemic waves were associated with walking, with participants reporting significantly higher levels of walking during the second pandemic wave compared to wave 4 (β = −0.19, *p* = 0.003), wave 5 (β = −0.22, *p* = 0.002) and waves 6–7 (β = −0.17, *p* = 0.028). COVID-19 waves represented the factor most strongly associated with walking levels during the third trimester, considering the standardized β-coefficients indicating a small-to-medium effect size. Further, living in a more densely populated area (β = 0.09, *p* = 0.005) and reporting a higher level of perceived partner support (β = 0.06, *p* = 0.044) were significantly associated with higher levels of walking. In addition, pregnant individuals with a university degree had higher levels of walking (β = 0.13, *p* = 0.019) compared to those with a high school diploma or less. Those who did not report pregnancy complications also reported higher levels of walking (β = −0.06, *p* = 0.039). Conversely, living with more children at home was associated with lower levels of walking in the third trimester of pregnancy (β = −0.16, *p* < 0.001). Again, standardized β-coefficients indicated small effect sizes for all of these associations [49]. No significant associations were found between other factors introduced in the model, such as COVID-19-related concerns and walking in the third trimester of pregnancy. As indicated by the R2 value, the model explained 8% of the variance in walking levels. For more details, see Table 4.

## 4. Discussion

### 4.1. Associations Between Walking Levels and Maternal Mental Health

The primary aim of this study was to examine associations between walking levels during the third trimester and four indices of prenatal maternal mental health in the context of the COVID-19 pandemic. Our findings contribute new knowledge, since studies focusing on walking during pregnancy [25,26] have mainly concentrated on depressive and anxiety symptoms without incorporating other relevant indices of prenatal maternal mental health—namely, perceived stress and pregnancy-related anxiety—and/or were not conducted during the COVID-19 pandemic. Our results showed that a higher level of walking in the third trimester was significantly associated with lower symptoms of generalized anxiety and perceived stress, after adjusting for covariates such as education level and perceived partner support. The standardized β-coefficients were even higher for perceived stress. Despite the association between walking and depression no longer being significant after adjusting for covariates, the effect size (β = −0.05) was similar to those obtained for the significant associations with generalized anxiety (β = −0.06) and perceived stress (β = −0.07). This is concordant with several studies reporting that a higher level of prenatal physical activity (PA) in general was associated with lower symptoms of depression and/or anxiety, whether studies were carried out during the pandemic [18], or before the pandemic [17,50].

However, it is relevant to note that effect sizes were small in our study. Pregnant individuals in our sample performed, on average, between 1 h and 2 h of slow walking (i.e., light-intensity PA) per week and between 30 min and 1 h of fast uphill walking (i.e., moderate-intensity PA) per week. National recommendations for pregnant individuals suggest at least 150 min (i.e., 2 h and 30 min) of moderate-intensity PA per week to obtain clinically meaningful health benefits [51]. It is not surprising that the time spent walking in our sample was generally lower than the weekly physical activity guideline of 150 min, since women may have engaged in other types of physical activities not captured by our study, such as swimming, especially during the 3rd trimester of pregnancy. As such, the walking duration in our sample may not have been sufficient to yield clinically significant effect, which could have resulted in greater reductions in mental health symptoms. Nevertheless, significant results were found despite the low duration of walking in our sample. From a public health perspective, our findings could suggest that within a pandemic context that has been shown in other studies to generate much stress and anxiety [5,6], even low duration of walking had the potential to support maternal mental health. Furthermore, although the reported intensity could not be objectively verified, additional path analyses (see Supplementary Materials) indicated that greater slow walking time only was associated with lower symptom levels across all maternal mental health indices, albeit with small effect sizes. Because participants reported a higher weekly duration of slow walking compared to fast uphill walking, these results could suggest that longer walking durations of lower intensity may confer greater benefits for maternal mental health than shorter walking durations of higher walking intensity. In other words, walking, especially slow walking, shows promising results to support mental health when other PA may not be accessible, either because of context (e.g., closures related to the pandemic) or personal situation (e.g., physical limitations due to the third trimester of pregnancy). However, to better understand walking’s potential as a public health strategy to promote maternal mental health, future studies should investigate whether longer walking durations are linked to greater symptom reductions and clinically meaningful improvements.

No significant association was initially observed between the total walking score and pregnancy-related anxiety symptoms in the third trimester. However, in the additional path analysis (see Supplementary Materials), where the walking variable was divided into two separate independent variables—slow walking scores and fast uphill walking scores—we found that only slow walking levels were associated with lower pregnancy-related anxiety symptoms after adjusting for covariates. To our knowledge, previous work has not studied associations between walking and pregnancy-related anxiety. In our study, the instrument used to assess pregnancy-related anxiety, the PRAQ-R2, addressed fears related to the moment of childbirth, but also fears about the health of the unborn baby and concerns about the mother’s physical appearance [11]. Slow walking, as a less physically demanding activity than fast walking, was associated with lower levels of pregnancy-related anxiety overall, like it was for the other three mental health indicators. Still, the potential mechanisms underlying these associations warrant further investigation to clarify the complex relationships between walking intensity and the different dimensions of pregnancy-related anxiety (e.g., concerns about physical appearance), as well as the direction of these links.

### 4.2. Factors Associated with Walking Levels During the Third Trimester

The second aim was to study factors associated with walking levels in the third trimester. Our results revealed that COVID-19 waves, population density, number of children at home, educational level, partner support, and pregnancy complications were associated with walking levels in the third trimester. To our knowledge, this is the first study to investigate factors associated with walking levels among pregnant individuals in the context of the COVID-19 pandemic.

We included the population density as a factor, which is relevant to consider, yet it has been neglected in previous studies examining walking among pregnant individuals during the pandemic. In our study, a more densely populated neighborhood of residence was associated with higher levels of walking in the third trimester of pregnancy during the COVID-19 pandemic. We had initially thought that individuals living in large, crowded cities might walk less due to the increased exposure to COVID-19 risks. However, in our study, COVID-19-related concerns and walking levels were not associated, so individuals who were more concerned about the risk of COVID-19 infection did not report lower levels of walking. Further, the more densely populated the environment, the more urban it becomes. Walking among adults is positively associated with areas that have a higher concentration of jobs, housing, and infrastructures [52,53]. It is possible that pregnant individuals in urban areas found it easier to incorporate walking into their daily activities, such as going to the grocery store, for example [24]. Moreover, consistent with our findings, one study found that pregnant individuals walked more in areas with better walkability [31]. The concept of walkability has gradually emerged to characterize places where walking is easier, due to greater safety and accessibility (e.g., availability of safe sidewalks and pedestrianized areas, street intersection density, proximity to public transit, diversity of employment, and occupied housing). Urban centers tend to have higher walkability scores, while rural areas generally score lower [30]. Living in a more walkable environment makes walking accessible, safer and more convenient by reducing the physical, time-related, and psychological barriers often associated with walking during pregnancy, whether it be for transportation, leisure, or health purposes [31]. It is, however, important to note that greater population density was associated with higher levels of generalized anxiety in our first path analysis. Indeed, although urban density may promote walking through greater walkability, it can also be associated with increased stress through factors such as noise, limited green spaces, crowding, and infection risk [54]. Policies at the neighborhood and city level should therefore aim to enhance factors that encourage walking while reducing stressors associated with high population density (e.g., providing safe, well-designed green spaces for walking) [55].

We also examined associations between COVID-19 waves and walking levels. We found that reported levels of walking were significantly higher during the second wave of COVID-19 (from 23 August 2020 to 20 March 2021) compared to waves 4, 5 and 6–7. The Quebec National Institute of Public Health has listed all events and government measures related to COVID-19 [32]. It can be seen from this timeline that the second wave was more restrictive in terms of government measures, with all regions rapidly moving to the red level (maximum alert). It is plausible that pregnant individuals had more time to dedicate to prenatal PA during wave 2, as many of the usual occupations of daily life (such as going to work in person, socializing activities) were suspended by government measures (e.g., most worked online, curfews were imposed, restaurant closures, etc.). Government measures became subsequently more flexible, particularly for vaccinated individuals, who were able to take advantage of the vaccine passport introduced at the start of wave 4. This passport gave them access to many of their usual activities [32]. Lack of time is a factor often cited to explain the low levels of prenatal PA among pregnant individuals [28]. However, we did not ask them directly why they did or did not engage in walking during each wave.

Further, the results of our study demonstrated that having more children at home was associated with lower levels of walking in the third trimester. Studies reveal that having children [56], especially under the age of five years [23], can prove to be a barrier to prenatal PA. Also, our results show that higher perceived partner support was significantly associated with higher levels of walking in the third trimester. Once again, the time factor could partly explain our results. Indeed, individuals with several children would potentially have less free time to devote to prenatal PA, including walking. Greater support at home may reduce the burden of daily responsibilities, allowing individuals to allocate more time to personal activities, including walking. In addition, pregnant individuals often cited encouragement and support from those around them as a factor facilitating prenatal PA [28].

We also found that individuals with a university degree reported higher levels of walking than individuals with a high school diploma or below. This result is also in accordance with previous findings concerning the link between educational levels and prenatal PA [22,23]. Finally, a significant association was found between a lower occurrence of pregnancy complications and higher levels of walking in the third trimester. Walking is considered a safe practice [24] and slow walking is not contraindicated even for those with severe complications, since maintenance of activities of daily living and domestic activities (light-intensity PA, including walking) is recommended [51]. However, the fear of severe complication (e.g., hemorrhage for placenta praevia) could explain the findings, even though it was not explicitly measured. In addition, pregnancy symptoms, such as pain and fatigue, are known to be an important reason why individuals express difficulty staying active [28]. Although individuals reported on pregnancy complications, we did not collect more detailed information on pain or discomfort specifically related to walking or other forms of prenatal PA.

### 4.3. Strengths, Limitations and Research Perspectives

Our study contributes novel findings to clarify our understanding of the association between walking and mental health of pregnant individuals in the context of the COVID-19 pandemic, by integrating four distinct indices of maternal mental health. It has also enabled us to identify new factors (e.g., population density, COVID-19 waves) associated with walking in a pandemic context. Pregnant individuals were recruited across multiple waves of COVID-19 and from various regions of Quebec, providing a diverse portrait of walking levels in late pregnancy within the Quebec pandemic context. The study also benefited from a large sample size (*n* = 1086).

However, this study faced several limitations. First, the use of self-reported measures to assess key variables, particularly walking, could have contributed to shared method variance. We used only two items to assess walking (i.e., slow walking and fast uphill walking), which may not have fully captured the range of walking activities performed by participants, such as fast walking on flat terrain or walking at varying speeds. Ideally, walking should be assessed using a combination of self-report and objective measures (e.g., pedometer, accelerometer, heart rate monitor). PA monitors could provide more accurate data on the real intensity and duration of walking. Second, the sample is not representative of the general population of pregnant individuals in Quebec since most of the participants were highly educated, had high family incomes, and predominantly identified as White. Third, recruitment was conducted primarily online, which likely led to a selection bias favoring individuals with higher socioeconomic status, greater digital access, and potentially more health awareness. Future studies should replicate these findings in more diverse and representative samples. Further, although the sample is drawn from a larger longitudinal study with multiple time points, our analyses focused on single data collection that does not allow us to infer the direction of the relationships observed between our variables of interest. For the path analysis and the multiple linear regression performed, the effect sizes of the explained variance—as reflected by the R2 values presented in the tables—remained small. This observation implies that other non-included and unmeasured variables could be associated with maternal mental health indices or with third trimester walking, and that their contribution may not have been considered in our models.

Confirming these associations through a walking-based intervention program could be a valuable next step. We focused on walking since it was the most accessible and popular activity among pregnant individuals. However, future studies could also examine walking in addition to other types of physical activity, such as prenatal yoga or swimming. Future follow-up with both the children and their mothers should be conducted and may further contribute to a deeper understanding of the clinical relevance of these findings.

## 5. Conclusions

The results indicate small yet significant associations between walking during the third trimester of pregnancy and some indicators of prenatal maternal mental health, following adjustment for relevant covariates. These findings highlight walking as a promising, accessible, safe, and low-cost public health strategy that could be promoted at the population level to support maternal physical and mental health. In addition, this study provides a better understanding of the factors associated with walking, guiding future research in the conceptualization and promotion of interventions targeting this form of PA. Such knowledge may help identify specific barriers to walking during pregnancy, inform strategies to effectively address them, and ultimately promote maternal mental health in stressful contexts such as a pandemic.

## Figures and Tables

**Table 1:** Descriptive statistics.

Variables	*n*	*N*
**Mother’s age (years)**		1059
18 to 27	232 (21.9%)	
28 to 35	682 (64.4%)	
36 to 45	145 (13.7%)	
**Ethnicity**		1068
White	1027 (96.2%)	
Black	11 (1%)	
Asian	11 (1%)	
Latin American	12 (1.1%)	
Other	7 (0.7%)	
**Education**		1055
High school diploma or less	60 (5.5%)	
Postsecondary diplomas (non-university)	306 (28.2%)	
University diploma	689 (65.3%)	
**Family income (CAD$)**		1046
49,999 or less	99 (9.5%)	
50,000 to 79,999	163 (15.6%)	
80,000 to 99,999	183 (17.5%)	
100,000 to 149,999	426 (40.7%)	
150,000 and more	175 (16.7%)	
**First pregnancy**		1080
Yes	592 (54.8%)	
No	488 (45.2%)	
**Pregnancy complications**		1086
No	834 (76.8%)	
Yes	252 (23.2%)	
**Number of children at home**		1080
0	653 (60.5%)	
1	297 (27.5%)	
2	98 (9.1%)	
3 or more	32 (2.9%)	
**Living with a partner**		1052
Yes	1009 (95.9%)	
No	43 (4.1%)	
**Density (number of people per km^2^)**		1065
400 or less (rural region)	541 (49.8%)	
401 and more (urban region)	524 (48.3%)	
**COVID-19 waves at completion**		1086
Second wave (23 August 2020–20 March 2021)	59 (5.4%)	
Third wave (21 March–17 July 2021)	63 (5.8%)	
Fourth wave (18 July–4 December 2021)	221 (20.3%)	
Fifth wave (5 December 2021–12 March 2022)	299 (27.5%)	
Sixth wave (13 March–28 May 2022)	207 (19.1%)	
Seventh wave (29 May 2022 onward)	237 (21.8%)	
**Season during completion**		1086
Winter	344 (31.7%)	
Spring	296 (27.3%)	
Summer	222 (20.4%)	
Fall	224 (20.6%)	

*Note*. Some participants did not answer all of the sociodemographic questions, which explains why the sample size may vary when considering each characteristic separately. Some variables, such as population density, number of children at home, and maternal age, were introduced in the analyses as continuous variables, but were categorized in this table to facilitate the interpretation of descriptive statistics.

**Table 2:** Descriptive statistics of maternal mental health indices and walking levels in the third trimester of pregnancy.

Variables	*N*	*Mean*	*SD*	*Min.*	*Max.*
**Maternal well-being indices**					
Generalized anxiety symptoms	1074	5.18	4.50	**0**	21
Perceived stress symptoms	1077	20.31	3.99	0	33
Depressive symptoms	1080	7.64	5.36	0	28
Pregnancy-related anxiety symptoms	1082	23.85	7.50	10	50
**Walking levels (METs-H/week)**					
Slow walking	1086	4.12	3.35	0	9.60
Uphill walking	1086	2.37	4.66	0	19.50
Total walking score	1086	6.50	6.75	0	29.10

**Table 3:** Results of the path analysis to study associations between walking levels and indices of prenatal maternal mental health during the third trimester of pregnancy.

Variables	*Generalized Anxiety*	*Depression*	*Perceived Stress*	*Pregnancy-Related Anxiety*
	β	β	β	β
Model 1				
Walking	−0.11 ***	−0.11 ***	−0.13 ***	−0.00
R^2^	0.01 *	0.01	0.02 *	0.00
Model 2				
Walking	−0.06 *	−0.05	−0.07 **	−0.02
Summer	−0.10	−0.10 *	−0.04	−0.10
Fall	−0.09	−0.07	−0.03	−0.03
Spring	−0.14 *	−0.11 *	−0.08	−0.13 *
Wave 2 of COVID-19	−0.06	−0.03	−0.03	−0.05
Wave 3 of COVID-19	0.04	0.05	0.05	−0.00
Wave 4 of COVID-19	−0.05	−0.04	−0.06	−0.08 *
Wave 5 of COVID-19	−0.03	0.01	−0.01	−0.13 *
Pregnancy complications	0.19 ***	0.16 ***	0.17 ***	0.13 ***
First pregnancy	0.04	0.04	0.07 *	−0.24 ***
University diploma	−0.19 **	−0.23 **	−0.08	−0.05
Postsecondary diplomas (non-university)	−0.11	−0.10	0.01	−0.04
Mother’s age	−0.13 ***	−0.05	−0.07 *	−0.02
Partner support	−0.27 ***	−0.34 ***	−0.33 ***	−0.18 ***
Population density	0.08 *	0.04	0.05	0.05
R^2^	0.17 ***	0.20 ***	0.19 ***	0.10 ***

*Note*. * *p* < 0.05 ** *p* < 0.01 *** *p* < 0.001. Two-tailed *p*-values < 0.05 were considered statistically significant. Dichotomous variables were coded as: first pregnancy: no = 1, yes = 2; Pregnancy complication: no = 0, yes = 1. It was possible to control for education level, seasons, and COVID-19 waves using dummy variables. For education level, the reference group was a high school diploma or less. For seasons, winter was the reference group. For COVID-19 waves, waves 6 and 7 combined constituted the reference group. Walking levels, mother’s age, partner support, and population density were continuous variables.

**Table 4:** Results of the multiple linear regression to study factors associated with walking levels during the third trimester.

Variables	β	*p*
Wave 3 of COVID-19	−0.07	0.123
Wave 4 of COVID-19	−0.19 **	**0.003**
Wave 5 of COVID-19	−0.22 **	**0.002**
Wave 6–7 of COVID-19	−0.17 *	**0.028**
Pregnancy complications	−0.06 *	**0.039**
Mother’s age	0.06	0.051
Number of children	−0.16 ***	**<0.001**
Partner support	0.06 *	**0.044**
Population density	0.09 **	**0.005**
COVID-19 concerns	−0.03	0.412
University diploma	0.13*	**0.019**
Postsecondary diplomas (non-university)	0.07	0.216
R^2^	0.08 ***	

*Note*. * *p* < 0.05 ** *p* < 0.01 *** *p* < 0.001. Two-tailed *p*-values < 0.05 were considered statistically significant. Dichotomous variables were coded as: Pregnancy complication: no = 0, yes = 1. It was possible to add education level and COVID-19 waves using dummy variables. For education level, the reference group was a high school diploma or less. For COVID-19 waves, wave 2 constituted the reference group. Mother’s age, number of children at home, partner support, population density, and COVID-19 concerns were continuous variables.

## Data Availability

The data presented in this study are available upon request following a data access protocol, due to ethical considerations. Requests to access the datasets should be directed to the corresponding author, C.M. Herba.

## Supplementary Materials

The following supporting information can be downloaded at: https://www.mdpi.com/article/10.3390/ijerph22101538/s1, Table S1: Associations between walking levels and indices of prenatal maternal mental health during the third trimester of pregnancy: separation of slow and fast walking.
