# Supplementary material for: Associations Between Walking in the Third Trimester of Pregnancy and Maternal Mental Health During the COVID-19 Pandemic
Source: Int J Environ Res Public Health. 2025 Oct 8;22(10):1538. doi: 10.3390/ijerph22101538 (PMC12563144; doi:10.3390/ijerph22101538)
Supplement: Supplementary file 1 [file ijerph-22-01538-s001.zip › ijerph-3819827-supplementary.pdf]

Supplementary Materials section

Table S1. Associations between walking levels and indices of prenatal maternal mental health during the third trimester of pregnancy: separation of slow and fast walking

	Generalized anxiety	*Depression*	*Perceived stress*	*Pregnancy-related anxiety*
**Variables**	β	β	β	β
Model 1				
Slow walking	**−0.11*** **	**−0.11*** **	**−0.10** **	**−0.09** **
Fast walking	−0.03	−0.03	−0.06	**0.07***
R^2	**0.02***	**0.02***	**0.02***	0.01
Model 2				
Slow walking	**−0.07***	**−0.06***	**−0.07***	**−0.10*** **
Fast walking	−0.01	0.00	−0.03	0.06
Summer	−0.10	−0.09	−0.03	−0.10
Fall	−0.09	−0.06	−0.02	−0.03
Spring	**−0.14***	**−0.11***	−0.08	**−0.12***
Wave 2 of COVID-19	−0.06	0.00	−0.02	−0.05
Wave 3 of COVID-19	0.04	0.05	0.05	0.00
Wave 4 of COVID-19	−0.05	−0.04	−0.06	**−0.08***
Wave 5 of COVID-19	−0.02	0.01	−0.01	**−0.12***
Pregnancy complications	**0.19*** **	**0.16*** **	**0.17*** **	**0.13*** **
First pregnancy	0.04	0.04	**0.07***	**−0.24*** **
University diploma	**−0.19***	**−0.22*** **	−0.08	−0.04
Postsecondary diplomas (non-university)	−0.11	−0.09	0.01	−0.03
Mother's age	**−0.12*** **	−0.05	**−0.06***	−0.02
Partner support	**−0.28*** **	**−0.34*** **	**−0.34*** **	**−0.18*** **
Population density	**0.08** **	0.05	0.06	0.05
R^2	**0.17*** **	**0.20*** **	**0.18*** **	**0.11*** **

Note. *$p<0.05$ ** $p<0.01$ ***$p<0.001$. Two-tailed *p*-values <.05 were considered statistically significant. Dichotomous variables were coded as: first pregnancy: no=1, yes =2; Pregnancy complication: no=0, yes=1. It was possible to control for education level, seasons, and COVID-19 waves using dummy variables. For education level, the reference group was a high school diploma or less. For seasons, winter was the reference group. For COVID-19 waves, waves 6 and 7 combined constituted the reference group. Walking levels, mother's age, partner support, and population density were continuous variables.
